# Deep Sleep, Olfactory Loss, and Cognition in Early-stage Parkinson’s Disease: Pilot Study Results

**DOI:** 10.1177/23337214241262925

**Published:** 2024-06-24

**Authors:** Vanessa M. Young, Rebecca Bernal, Erin Pollet, Luis Serrano-Rubio, Carlos Gaona, Jayandra Jung Himali, Sudha Seshadri, David Andrés González, Mitzi M. Gonzales

**Affiliations:** 1 Glenn Biggs Institute for Alzheimer’s and Neurodegenerative Diseases, University of Texas Health Science Center at San Antonio, USA; 2 Arizona State University, Phoenix, USA; 3 Academy Diagnostics, San Antonio, TX, USA; 4 Boston University School of Public Health, MA, USA; 5 Boston University School of Medicine, MA, USA; 6 Rush University Medical Center, Chicago, IL, USA; 7 Cedars Sinai Medical Center, Los Angeles, CA, USA

**Keywords:** cognition, sleep disorders, Parkinson’s disease, aging

## Abstract

Individuals with Parkinson’s disease (PD) have a higher risk of developing dementia compared to age-matched controls. Rapid eye movement sleep behavior disorder (RBD) and hyposmia can influence symptoms severity. We report associations between polysomnography-assessed sleep architecture, olfactory identification, and cognition. Twenty adults with early-stage PD (mean age 69 ± 7.9; 25% female) completed cognitive assessments, the Brief Smell Identification Test (BSIT), and overnight in-clinic polysomnography. A global cognitive score was derived from principal component analysis. Linear regression models examined associations between sleep variables, BSIT performance, and cognition. Cognitive performance was compared between participants with and without RBD. Deep sleep attainment (β ± SE: 1.18 ± 0.45, *p* = .02) and olfactory identification (0.37 ± 0.12, *p* = .01) were associated with better cognition. Light sleep, REM sleep, arousal index, and sleep efficiency were not (all *p* > .05). Participants with RBD had significantly worse cognition (*t*-test = −1.06 ± 0.44, *p* = .03) compared to those without RBD; none entered deep sleep. Deep sleep attainment was associated with better memory (1.20 ± 0.41, *p* = .01) and executive function (2.94 ± 1.13, *p* = .02); sleep efficiency was associated with executive function (0.05 ± 0.02, *p* = .02). These findings suggest interrelationships between lack of deep sleep, hyposmia, and poorer cognition in PD, particularly among individuals with RBD. Assessing these markers together may improve early identification of high-risk individuals and access to interventions.

## Introduction

The risk of developing dementia is 2.5 to 6 times higher in individuals with Parkinson’s disease (PD) compared to age-matched controls without PD (Aarsland et al., 2021). In PD, cognitive decline typically emerges first in domains governed by fronto-subcortical networks such as executive functioning and processing speed, following the onset of motor symptoms (Aarsland et al., 2021). The severity of motor and non-motor impairments, as well as the rate of disease progression, are variable and can be influenced by factors such as medication regimen, presence of sleep disturbances like rapid eye movement sleep behavior disorder (RBD), hyposmia, and genetic mutations like those in the GBA1 gene (GBA1-associated PD) (Aarsland et al., 2021; Haehner et al., 2019; Kang et al., 2016; Roeben et al., 2024). Gaining a deeper understanding of biomarkers that predict individual cognitive trajectories in Parkinson’s disease may better inform prognosis and support the development of tailored interventions.

At least 66% of individuals with PD experience sleep disturbances with RBD as a primary manifestation (Aarsland et al., 2021; Haehner et al., 2019). Idiopathic RBD is considered a prodromal syndrome of alpha-synuclein neurodegeneration, the neuropathological protein associated with PD (Haehner et al., 2019). RBD is linked to accelerated cognitive decline, suggesting that it may reflect a subtype of PD with more widespread cortical degeneration (Haehner et al., 2019). Another potential harbinger of PD is hyposmia, which relates to cholinergic denervation and frequently emerges decades before motor symptom onset (Aarsland et al., 2021; Kang et al., 2016). Among individuals with PD, RBD and hyposmia have both been linked to more severe motor and cognitive dysfunction (Aarsland et al., 2021; Roguski et al., 2022). In addition, the presence of RBD in conjunction with hyposmia was associated with poorer cognitive performance (Kang et al., 2016), stressing their importance for risk detection and understanding underlying disease biology. Further reading on RBD among those with PD can be found in the supplementary SIntroduction. Herein, we comprehensively and objectively assessed sleep architecture, hyposmia and multi-domain cognition together.

Understanding the interrelationships between sleep, hyposmia and cognition is critical as they each have been independently associated with worse PD prognosis (Aarsland et al., 2021; Haehner et al., 2019; Kang et al., 2016; Roguski et al., 2022). We report associations between polysomnography-assessed sleep architecture, olfactory sensitivity, and cognition in individuals with early-stage PD.

## Methods

This study reports on secondary analysis from the prospective, cross-sectional study named, *Sleep Issues – Efficacy and Sensitivity of Technological Assessments* (SIESTA) (Gonzales et al., 2023). The SIESTA study was conducted according to the World Medical Association Declaration of Helsinki. The IRB at University of Texas Health Science at San Antonio (UTHSCSA) approved the study procedures and all participants provided written informed consent.

### Sample

Participants were recruited from the Department of Neurology at UTHSCSA between January and November 2021. Eligible participants included adults (age range 18–88 years) with a clinical diagnosis of PD with and without RBD and the availability of a study partner capable of providing collateral data on sleep. *Exclusion criteria* included the following diagnoses: (1) insomnia or a score > 21 in the Insomnia Severity Index (Bastien et al., 2001); sleep apnea or a score > 2 in the STOP-BANG (Chung et al., 2008) if untreated; (2) restless leg syndrome if untreated; and (3) pre-existing clinical dementia diagnosis or a score < 19 in the Montreal Cognitive Assessment (Hoops et al., 2009). In addition, those with a body mass index (BMI) score >39 kg/m^2^ or using sedative-hypnotic medications (benzodiazepines, benzodiazepine receptor agonists, pregabalin, and antipsychotics) within 2 weeks preceding and during study participation were *excluded*. Due to the small sample size, participants with an AHI of ≥5 were not excluded.

### Measures

#### Rapid Eye Movement Sleep Behavior Disorder

RBD was evaluated through clinical interview and completion of questionnaires. Participants completed the RBD Single Question Screen (RBD1Q), and study partners completed the Mayo Sleep Questionnaire. Both questionnaires have been validated against PSG for detecting RBD and have acceptable sensitivity (93%–100%) and specificity (87%–95%).

#### Cognition

A trained research assistant administrated a multidomain cognitive battery following standardized administration procedures during participants’ first visit at the clinic. The cognitive battery assessed memory (Hopkins Verbal Learning Test-Revised; Brief Visuospatial Memory Test—Revised), executive function (Trail Making Test Part B, Category and Phonemic Fluency), and processing speed (Trail Making Test Part A; Oral Symbol Digit Modalities Test [SDMT]) (Lezak et al., 2012). Details about each individual test can be found in Supplemental Methods and Table S1.

#### Olfactory Identification

The Brief Smell Identification Test (BSIT) evaluated olfactory identification (Doty et al., 1996) during their first visit at the clinic. The BSIT comprises 12 different odorants embedded on small strips of paper. Participants were given a multiple-choice questionnaire with four answer options to identify each of the 12 smells. Higher score indicates better olfactory identification performance. Based on the manual, participants can be categorized as having either normal (B-SIT score of ≥9) or abnormal (B-SIT score < 9) olfactory function, irrespective of sex. Raw scores on the BSIT were used for the current analysis.

#### Polysomnography

All participants completed an overnight polysomnography (PSG) within 4 weeks of neuropsychological assessment, which involved comprehensive monitoring of various physiological signals. Specifically, electroencephalographic (EEG) activity was recorded from frontal, central, and occipital regions, along with electrooculographic (EOG) measurements of eye movements and submental electromyographic (EMG) recordings of chin muscle activity. Respiratory parameters such as nasal and oral airflow, anterior tibialis electromyography (to assess leg movements), snoring sounds via a microphone, body position, and electrocardiographic data were also acquired. Thoracic and abdominal excursions were measured using inductance plethysmography, allowing for the assessment of respiratory effort. Moreover, oxygen saturation levels (SpO2) were continuously monitored through pulse oximetry, providing insights into potential oxygen desaturation events during sleep. In accordance with American Academy of Sleep Medicine criteria version 2.5, the scored epochs were categorized into one of the five sleep stages: wake (W), REM, non-REM stage1 (N1), non-REM stage 2 (N2), and non-REM stage 3 (N3). For this analysis, sleep stages were examined as percentages of total sleep time (TST). Percent of TST spent in stages N1 and N2 were summed to reflect light sleep, and percent of TST in N3 reflecting slow wave sleep represented deep sleep.

### Statistical Analyses

Percentage of TST in REM sleep and the arousal index were normalized using square root and natural log transformations, respectively. Light sleep was dichotomized using a median split (cut off point = 91.40) and percentage of TST in deep sleep was binarized as any amount (*n* = 6) versus none (*n* = 14). Demographic and clinical variables were assessed using descriptive statistics. Cognitive test scores were reversed as needed so higher scores indicated better performance; non-normally distributed data was transformed using log. The primary outcome, global cognition, was derived by applying principal component analysis (PCA) to all cognitive assessments listed in the Methods section and forcing a single-factor solution (Pase et al., 2023) (Supplemental Methods and Table S1).

Cognitive domains of memory, executive function, and processing speed abilities were assessed as secondary outcomes and were derived using composite *z*-scores (Supplemental Methods and Table S1). For each raw cognitive variable, a *z*-score was calculated for each subject by subtracting the mean and dividing by the standard deviation of the full study population. A domain *z*-score was then created by summing the *z*-scores for all tests in that domain, subtracting the domain mean, and dividing by the domain standard deviation. For these analyses, *z*-scores were signed so higher scores indicate better performance.

Associations between each sleep variable, BSIT raw scores, and cognition were measured with unadjusted (model 1) and age plus age-squared adjusted (model 2) regression analyses. Sex and education were not statistically significant covariates. Differences between the RBD and non-RBD groups were assessed using independent *t*-tests. A two-sided *p* < .05 was considered to be statistically significant. We utilized SAS statistical software version 9.4 (SAS Institute) to run the analyses, excluding missing variables analysis by analysis.

## Results

Twenty adults with early-stage PD (Hoehn and Yahr Stage 1–2) and without dementia were included. The average age was 69 ± 7.9; 25% of the sample was female, and 50% reported having a college degree (see Supplemental Table 2). Eighteen participants were included in the PCA due to missing data on neuropsychological tests on two participants. Of these 18 participants, 11 (61%) met criteria for RBD and endorsed experiencing RBD symptoms at least once monthly. As displayed in Table 1, deep sleep attainment and BSIT performance were associated with better global cognition in the unadjusted model. After adjusting for aged squared, BSIT performance but not deep sleep remained associated with better global cognition. Sleep efficiency, arousal index, percentages of light sleep and REM sleep (see Figure S1) were not significantly associated with global cognition in the unadjusted and adjusted models, yet the direction of the relationships remains consistent with results from larger studies (Pase et al., 2023). In our sample, none of the participants with RBD entered deep sleep and only 7 out of 12 entered REM sleep. Furthermore, RBD diagnosis was associated with worse global cognition (*t*-test = −1.06 ± 0.44, *p* = .03; Figure S3). Both Deep sleep and sleep efficiency were associated with BSIT score in the unadjusted model only.

**Table 1.** Associations Between Sleep Architecture, Cognition, and Olfaction.

		Light sleep^a^			Deep sleep^b^			REM sleep^c^			Arousal index^d^			Sleep efficiency		
Outcomes	Models	β	SE	*p*	β	SE	*p*	β	SE	*p*	β	SE	*p*	β	SE	*p*
Primary outcomes																
Global cognition (*n* = 18)	1	–.46	0.47	.35	**1.18**	**0.45**	**.02**	.06	0.12	.62	.17	0.59	.77	.02	0.01	.12
	2	.32	0.45	.49	.97	0.53	.09	.07	0.12	.55	–.06	0.55	.91	.02	0.01	.10
BSIT (0–12) (*n* = 19)	1	–1.60	0.92	.10	**.73**	**0.29**	**.02**	.27	0.24	.29	–.05	1.59	.98	**.04**	**0.02**	**.04**
	2	1.20	1.08	.28	2.58	1.29	.06	.17	0.27	.53	.09	1.62	.96	.03	0.02	.10
Secondary outcomes																
Memory (*n* = 20)	1	–.65	0.43	.15	**1.20**	**0.41**	**.01**	.11	0.11	.33	.26	0.59	.66	.01	0.01	.19
	2	.41	0.41	.34	.83	0.50	.12	.08	0.10	.42	–.01	0.52	.99	.01	0.01	.29
Executive function (*n* = 19)	1	–1.07	1.15	.37	**2.94**	**1.13**	**.02**	.18	0.30	.56	–.44	1.46	.77	**.05**	**0.02**	**.02**
	2	.81	1.17	.50	2.75	1.37	.06	.19	0.29	.51	–1.00	1.41	.49	**.05**	**0.02**	**.01**
Processing speed (*n* = 18)	1	–.59	0.74	.44	1.37	0.77	.09	.12	0.19	.53	.19	0.92	.84	.02	0.01	.24
	2	.32	0.78	.69	.98	0.97	.33	.10	0.20	.61	.03	0.93	.97	.02	0.02	.32

*Note.* β ± *SE* and *p*-values derived from linear regression models examining the associations between sleep with cognition and odor identification. Model 1 was unadjusted. Model 2 was adjusted for age and age squared.

a Dichotomized as <91.40% or >91.40% of total sleep time.

b Dichotomized as 0% or >0% percentage of total sleep time.

c Percentage of total sleep time with square root transformation applied.

d Natural log transformed.

Significant findings at *p* < .05 are shown in bold.

Secondary analyses of cognitive domains revealed deep sleep attainment was associated with better memory and executive function in the unadjusted model; whereas sleep efficiency was associated with executive function in both models. Finally, BSIT performance were associated with better global cognition in the unadjusted mode (0.37 ± 0.12, *p* = .01; Figure S3). After adjusting for aged squared, BSIT performance remained associated with better global cognition (0.34 ± 0.10, *p* < .001). Table 1 presents adjusted and unadjusted results for primary and secondary analyses.

## Discussion

This preliminary investigation revealed notable associations between deep sleep, olfactory function, and cognitive performance in PD. The intersectionality of these highly prevalent symptoms is not sufficiently studied; our exploratory results point to readily measurable markers that could potentially improve risk stratification, timing of interventions, and understanding of pathogenesis. Despite the limited statistical power, the association between deep sleep and global cognition remained a key observation. Notably, none of the participants with RBD entered deep sleep and 7 out of 12 did not enter REM sleep; the RBD group showed significantly worse cognitive performance compared to the non-RBD group. This aligns with prior evidence that RBD may be a biomarker of accelerated cognitive decline and more rapid disease progression (Roguski et al., 2022). In parallel, olfactory sensitivity associated with deep sleep attainment. The relationship between olfaction and deep sleep deficit provides preliminary evidence for an inter-relationship between disturbed sleep and hyposmia in PD. Importantly, the results suggest that interventions to increase deep sleep may potentially offset cognitive decline in PD.

While promising, limitations include the binary deep sleep measure lacking nuanced slow-wave quantification and the lack of performance validity measures in the cognitive battery. Further, we were unable to examine REM sleep without atonia as the majority of participants with RBD did not enter REM sleep, and our PSG metrics did not include tonic REM, phasic REM, and REM sleep without atonia percentages. Future investigations should incorporate these parameters, as they may reveal novel relationships and underlying mechanisms in the clinical realm of RBD. Longitudinal tracking of comprehensive sleep and smell measures in relation to cognitive change are needed to confirm these observations and elucidate the directionality and causal nature of the associations. Replication in larger, more diverse PD cohorts with adequate RBD and non-RBD representation is essential for increasing generalizability.

In summary, these preliminary findings suggest complex interrelationships between lack of deep sleep, hyposmia, and poorer cognition in PD, particularly among those with RBD. Assessing these markers together may improve early detection and monitoring of PD progression, as well as aiding in the identification of high-risk populations who may benefit from tailored interventions. Longitudinal multi-modal imaging and CSF biomarker studies are warranted to elucidate the complex neurobiological mechanisms linking alpha-synuclein pathology, cholinergic denervation, and neurodegeneration across brain regions.

## Supplemental Material

sj-docx-1-ggm-10.1177_23337214241262925 – Supplemental material for Deep Sleep, Olfactory Loss, and Cognition in Early-stage Parkinson’s Disease: Pilot Study Results Supplemental material, sj-docx-1-ggm-10.1177_23337214241262925 for Deep Sleep, Olfactory Loss, and Cognition in Early-stage Parkinson’s Disease: Pilot Study Results by Vanessa M. Young, Rebecca Bernal, Erin Pollet, Luis Serrano-Rubio, Carlos Gaona, Jayandra Jung Himali, Sudha Seshadri, David Andrés González and Mitzi M. Gonzales in Gerontology and Geriatric Medicine

## References

[bibr1-23337214241262925] Aarsland D. Batzu L. Halliday G. M. Geurtsen G. J. Ballard C. Ray Chaudhuri K. Weintraub D. (2021). Parkinson disease-associated cognitive impairment. Nature Reviews Disease Primers, 7(1), 47. 10.1038/s41572-021-00280-3 34210995

[bibr2-23337214241262925] Bastien C. H. Vallières A. Morin C. M. (2001). Validation of the insomnia severity index as an outcome measure for insomnia research. Sleep Medicine, 2(4), 297–307. 10.1016/s1389-9457(00)00065-4 11438246

[bibr3-23337214241262925] Chung F. Yegneswaran B. Liao P. Chung S. A. Vairavanathan S. Islam S. Khajehdehi A. Shapiro C. M. (2008). STOP questionnaire: A tool to screen patients for obstructive sleep apnea. Anesthesiology, 108(5), 812–821. 10.1097/ALN.0b013e31816d83e4 18431116

[bibr4-23337214241262925] Doty R. L. Marcus A. Lee W. W. (1996). Development of the 12-item cross-cultural smell identification test (CC-SIT). Laryngoscope, 106(3 Pt 1), 353–356. 10.1097/00005537-199603000-00021 8614203

[bibr5-23337214241262925] Gonzales M. M. Wang D. Pollet E. Velarde Á Horn S. Coss P. Vaou O. Wang J. Li C. Seshadri S. Miao H. González D. A. (2023). Pilot Study Results Assessing the Accuracy of a Ballistic Sleep Monitor Relative to Polysomnography in Parkinson’s Disease. Journal of Parkinson’s disease, 13(6), 1073–1076. 10.3233/JPD-23012 PMC10578264 37599538

[bibr6-23337214241262925] Haehner A. Masala C. Walter S. Reichmann H. Hummel T. (2019). Incidence of Parkinson’s disease in a large patient cohort with idiopathic smell and taste loss. Journal of Neurology, 266(2), 339–345. 10.1007/s00415-018-9135-x 30488238

[bibr7-23337214241262925] Hoops S. Nazem S. Siderowf A. D. Duda J. E. Xie S. X. Stern M. B. Weintraub D. (2009). Validity of the MoCA and MMSE in the detection of MCI and dementia in Parkinson disease. Neurology, 73(21), 1738–1745. 10.1212/WNL.0b013e3181c34b47 19933974 PMC2788810

[bibr8-23337214241262925] Kang S. H. Lee H. M. Seo W.-K. Kim J. H. Koh S.-B. (2016). The combined effect of REM sleep behavior disorder and hyposmia on cognition and motor phenotype in Parkinson’s disease. Journal of Neurological Sciences, 368, 374–378. 10.1016/j.jns.2016.07.057Scopus. 27538667

[bibr9-23337214241262925] Lezak M. D. Howieson D. B. Bigler E. D. Tranel D. (Eds.). (2012). Neuropsychological assessment (5th ed., p. 1161). Oxford University Press.

[bibr10-23337214241262925] Pase M. P. Harrison S. Misialek J. R. Kline C. E. Cavuoto M. Baril A.-A. Yiallourou S. Bisson A. Himali D. Leng Y. Yang Q. Seshadri S. Beiser A. Gottesman R. F. Redline S. Lopez O. Lutsey P. L. Yaffe K. Stone K. L. . . . Himali J. J. (2023). Sleep Architecture, obstructive sleep apnea, and cognitive function in adults. JAMA network open, 6(7), e2325152. 10.1001/jamanetworkopen.2023.25152 PMC10354680 37462968

[bibr11-23337214241262925] Roeben B. Liepelt-Scarfone I. Lerche S. Zimmermann M. Wurster I. Sünkel U. Schulte C. Deuschle C. Eschweiler G. W. Maetzler W. Gasser T. Berg D. Brockmann K. (2024). Longitudinal cognitive decline characterizes the profile of non-PD-manifest GBA1 mutation carriers. npj Parkinson’s Disease, 10(1), 88–89. 10.1038/s41531-024-00706-1 PMC11035543 38649346

[bibr12-23337214241262925] Roguski A. Rolinski M. Jones M. W. Whone A. (2022). Inaccurate self-report of olfactory dysfunction in REM sleep behaviour disorder and implications for prognosis. Clinical Parkinsonism and Related Disorders, 8, 100176. 10.1016/j.prdoa.2022.100176 PMC9804136 36594073

