# Supplementary material for: Deep Sleep, Olfactory Loss, and Cognition in Early-stage Parkinson’s Disease: Pilot Study Results
Source: Gerontol Geriatr Med. 2024 Jun 24;10:23337214241262925. doi: 10.1177/23337214241262925 (PMC11265233; doi:10.1177/23337214241262925)
Supplement: sj-docx-1-ggm-10.1177_23337214241262925 – Supplemental material for Deep Sleep, Olfactory Loss, and Cognition in Early-stage Parkinson’s Disease: Pilot Study Results [file sj-docx-1-ggm-10.1177_23337214241262925.docx]

**Supplemental Online Content**

**SIntro.** Further Reading on Select Topics.

**SMethods.** Cognitive Battery.

**Table S1.** Cognitive Tasks used in Computing Global and Domain-specific Cognitive Composite Scores

**Table S2.** Demographic and Clinical Characteristics the Sample.

**FigureS1.** Association Between REM Sleep and Global Cognition

**FigureS2.** Association Between Brief Smell Identification Score and Global Cognition

**FigureS3.** Global Cognition by REM Sleep Behavior Disorder Diagnosis Status

**FigureS4.** Smell Identification Performance by REM Sleep Behavior Disorder Diagnosis Status

**SIntroduction**

Given space and reference constraints, we have added a section for further reading on select topics.

**Parkinson Disease & Rapid Eye Movement Sleep Behavior Disorder**

Galbiati, A., Verga, L., Giora, E., Zucconi, M., & Ferini-Strambi, L. (2019). The risk of neurodegeneration in REM sleep behavior disorder: a systematic review and meta-analysis of longitudinal studies. *Sleep medicine reviews*, *43*, 37-46.

Gros, P., & Videnovic, A. (2020). Overview of sleep and circadian rhythm disorders in Parkinson disease. *Clinics in geriatric medicine*, *36*(1), 119-130.

Van Patten, R., Mahmood, Z., Pickell, D., Maye, J. E., Roesch, S., Twamley, E. W., ... & Schiehser, D. M. (2022). REM Sleep Behavior Disorder in Parkinson’s Disease: Change in Cognitive, Psychiatric, and Functional Outcomes from Baseline to 16–47-Month Follow-Up. *Archives of Clinical Neuropsychology*, *37*(1), 1-11.

Xie, C., Zhu, M., & Hu, Y. (2021). Risk stratification for REM sleep behavior disorder in patients with Parkinson’s disease: A PRISMA-compliant meta-analysis and systematic review. *Clinical Neurology and Neurosurgery*, *202*, 106484.

**Parkinson Disease & Cognition**

Aarsland, D., Batzu, L., Halliday, G. M., Geurtsen, G. J., Ballard, C., Ray Chaudhuri, K., & Weintraub, D. (2021). Parkinson disease-associated cognitive impairment. *Nature Reviews Disease Primers*, *7*(1), 1-21.

Magnante, A. T., Ord, A. S., Holland, J. A., & Sautter, S. W. (2022). Neurocognitive functioning of patients with early-stage Parkinson’s disease. *Applied Neuropsychology: Adult*, 1-12.

Saredakis, D., Collins-Praino, L. E., Gutteridge, D. S., Stephan, B. C., & Keage, H. A. (2019). Conversion to MCI and dementia in Parkinson's disease: a systematic review and meta-analysis. *Parkinsonism & Related Disorders*, *65*, 20-31.

Wallace, E. R., Segerstrom, S. C., van Horne, C. G., Schmitt, F. A., & Koehl, L. M. (2022). Meta-analysis of cognition in Parkinson’s disease mild cognitive impairment and dementia progression. *Neuropsychology review*, 1-12.

**SMethods**

**Cognitive Battery**

*Hopkins Verbal Learning Test-Revised* *(HVLT-R)*

The HVLT-R (Benedict et al., 1998) is a verbal memory test that involves learning a list of 12 words grouped into 3 semantic categories over three learning trials. Participants were then asked to recall the word list after a delay of around 20 to 25 minutes. The Total Immediate Recall score represents the total number of correctly recalled words across the three learning trials, while the Delayed Recall Score measures the number of words correctly recalled during the delayed trial.

*Trail Making Tests A and B (TMT-A and TMT-B)*

*TMT-A and TMT-B* (Reitan, 1958) are commonly used assessments that measure attention, processing speed, and executive functioning. TMT-A consists of connecting 25 numbered circles in sequence, reflecting visuomotor tracking and processing speed, while TMT-B involves a more complex task that assesses cognitive flexibility, working memory, and other executive functions. Time completion is assessed in seconds; wherein, longer completion time indicates worse performance Herein, the completion time was reversed, and longer completion time indicates better performance (Table S1).

*Brief Visuospatial Memory Test-revised (BVMT-R)*

The BVMT-R (Benedict, 1997) is a nonverbal memory test where participants studied a card with 6 geometric figures for 10 seconds and then reproduced them in their correct locations. Participants underwent 3 learning trials, and they were asked to draw the figures again after about 20 to 25 minutes. The Total Immediate Recall score indicates the total number of correctly reproduced figures from all 3 trials, while the Delayed Score reflects the number of figures correctly reproduced during the delayed trial.

*Category and Phonemic Fluency*

To assess Category Fluency, participants were asked to generate words belonging to the categories of animals and vegetables. Phonemic Fluency was measured by instructing participants to generate words beginning with the letters F and L. Participants had one minute for each task to provide responses, which were recorded verbatim.

*Symbol Digit Modalities Test (SDMT)*

The SDMT (Smith, 2016) measures the speed of processing information. Participants were presented with a sheet containing nine symbols paired with numbers and are required to verbally match each symbol with its corresponding number within a time limit of 90 seconds. The outcome variable gauged is the total number of correct responses within this timeframe.

**Table S1**

*Cognitive Tasks and Creation of Global and Domain-specific Cognitive Composite Scores*

| **Cognitive Tasks** | **Transformation** | **Standardizing Formula** | **Component Loading** |
| --- | --- | --- | --- |
| **Global Cognitive Score** |  |  |  |
| *HVLTR (Delay + Recall), N correct* |  | (HVLTRc + 0.03)/1.033 | 0.20 |
| *BVMT (Delay + Recall), N correct* |  | (BVMTc+0.01)/0.96 | 0.19 |
| *Trail A, seconds* | - log (TMT-A) | (TRAILA-0.01)/1.03 | 0.21 |
| *Trail B, seconds* | - log (TMT-B) | (TRAILB-0.04)/0.93 | 0.26 |
| *SDMT, N correct* |  | (SDMT-0)/1 | 0.16 |
| *Category Fluency, N correct* |  | (CatFluency-0.03)/1.02 | 0.22 |
| *Phonemic Fluency, N correct* |  | (PhFluency+0.03)/1.05 | 0.16 |
| **Executive Function** |  |  |  |
| *Category Fluency, N correct* |  | Z scored |  |
| *Phonemic Fluency, N correct* |  | Z scored |  |
| *Trail B, seconds* |  | Z scored |  |
| **Memory** |  |  |  |
| *HVLTR (Delay + Recall), N correct* |  | Z scored |  |
| *BVMT (Delay + Recall), N correct* |  | Z scored |  |
| **Processing Speed** |  |  |  |
| *Trail A, seconds* |  | Z scored |  |
| *SDMT, N correct* |  | Z scored |  |

*Note*. HVLTR: Hopkins Verbal Learning Test -Revised; BVMT: Brief Visuospatial Memory Test; Symbol Digit Modalities Test (SDMT)

**Table S2.** Demographic and Clinical Characteristics of Whole Sample.

|  |  | N=20 |
| --- | --- | --- |
| Age, years, mean (SD) | 69 | (7.9) |
| Female, N (%) | 5 | (25%) |
| Race/Ethnicity, N (%) |  |  |
| *Non-Hispanic White* | 18 | (90%) |
| *Hispanic* | 1 | (5%) |
| *Black* | 1 | (5%) |
| Education, N (%) |  |  |
| *Some College* | 3 | (15%) |
| *College Graduate* | 10 | (50%) |
| *Post-graduate* | 7 | (35%) |
| REM Sleep Behavioral Disorder Diagnosis, N (%) | 12 | (60%) |
| Sleep Stages, mean (SD) |  |  |
| *Percent Light Sleep* | 90.89 | (10.1) |
| *Percent Deep Sleep* | 0.64 | (1.5) |
| *Percent REM Sleep* | 8.49 | (9.5) |
| Sleep Efficiency, mean (SD) | 55.6 | (26.6) |
| Arousal Index, ≥5, N (%) | 20 | (100%) |
| Brief Smell Identification Test, mean (SD) | 5.84 | 2.1 |
| *Normal (score > 9), N (%)* | 2 | (10.53%) |
| *Abnormal -hyposmia (score≤ 9), N (%)* | 17 | (89.47%) |

**FigureS1. Association Between REM Sleep and Global Cognition**


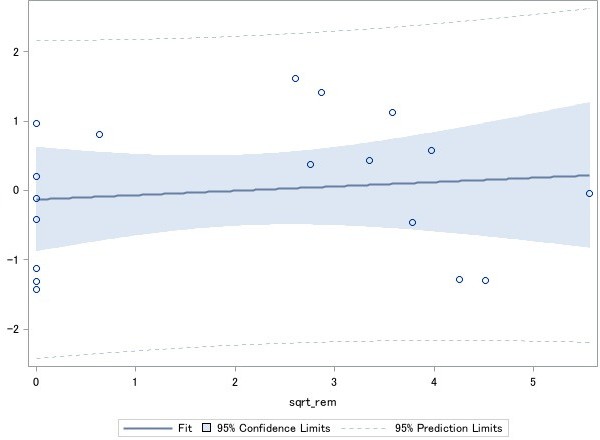


**REM Sleep**

**Global Cognition**

**FigureS2. Association Between Brief Smell Identification Score and Global Cognition**


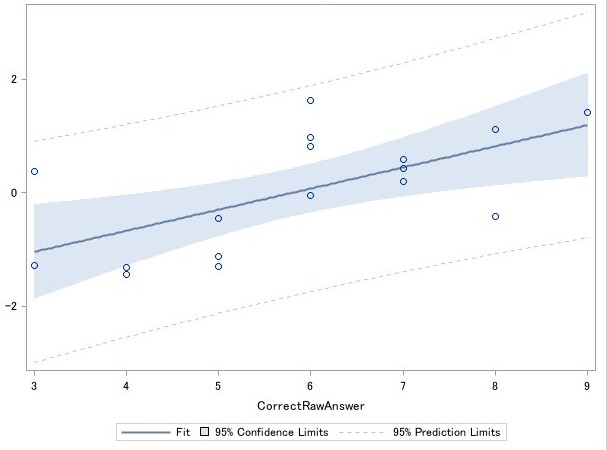


**Brief Smell Identification Test**

**Global Cognition**

**FigureS3. Global Cognition by REM Sleep Behavior Disorder Diagnosis Status**


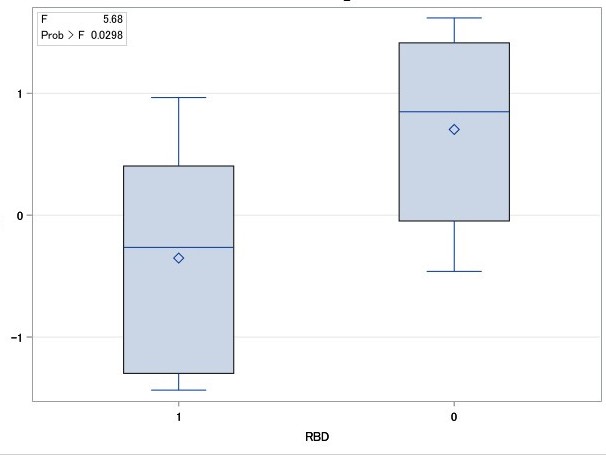


**RBD**

**non-RBD**

**Global Cognition**

**FigureS4. Smell Identification Performance by REM Sleep Behavior Disorder Diagnosis Status**


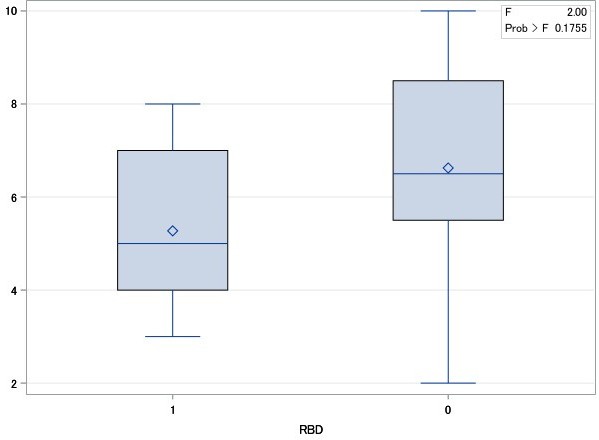


**RBD**

**non-RBD**

**Global Cognition**

**Supplementary References**

Benedict, R. H. B. (1997). *Brief Visuospatial Memory Test—Revised*. PAR.

Benedict, R. H. B., Schretlen, D., Groninger, L., & Brandt, J. (1998). Hopkins Verbal Learning Test – Revised: Normative Data and Analysis of Inter-Form and Test-Retest Reliability. *The Clinical Neuropsychologist*, *12*(1), 43–55. https://doi.org/10.1076/clin.12.1.43.1726

Reitan, R. M. (1958). Validity of the Trail Making Test as an Indicator of Organic Brain Damage. *Perceptual and Motor Skills*, *8*(3), 271–276. https://doi.org/10.2466/pms.1958.8.3.271

Smith, A. (2016). *Symbol Digit Modalities Test* [dataset]. https://doi.org/10.1037/t27513-000
